# Effects of HMGA2 on the epithelial-mesenchymal transition-related genes in ACHN renal cell carcinoma cells-derived xenografts in nude mice

**DOI:** 10.1186/s12885-022-09537-w

**Published:** 2022-04-19

**Authors:** Ying Liu, Guangyao Lv, Jianxin Bai, Lingling Song, Elizabeth Ding, Lin Liu, Yuqin Tian, Qian Chen, Kai Li, Xianfeng Liu, Yan Ding

**Affiliations:** 1 Department of Urology, Affiliated Zhongshan Hospital of Dalian University, Dalian, China; 2 Department of Intervention, The Second Affiliated Hospital of Dalian Medical University, Dalian, China; 3 Department of Neuroscience, Brown University, Providence, RI 02912 USA; 4 Navy Qingdao Special Care Center, Qingdao, 266071 China; 5 Department of Surgical Operations, Affiliated Hospital of Qingdao University, Qingdao, China; 6 The Institute for Translational Medicine Affiliated Zhongshan Hospital of Dalian University, Dalian, 116001 China; 7 Department of Pediatrics, Children’s Hospital of Boston, Harvard Medical School, Boston, MA 02115 USA

**Keywords:** HMGA2, shRNA, Epithelial-mesenchymal transition, ACHN cells, Xenograft

## Abstract

**Background:**

The architectural transcriptional regulator high-mobility group AT-hook 2 (HMGA2) is an oncofetal protein which has been reported to be ectopically expressed in a variety of cancers. A high expression of HMGA2 in human renal cell carcinoma (RCC) is related with tumor invasiveness and poor prognosis. Recent in vitro studies have shown that HMGA2 knockdown was able to decrease cell proliferation and migration, and regulate the gene expression related to epithelial-mesenchymal transition (EMT).

**Methods:**

To understand the HMGA2’s effect in vivo, HMGA2 expression was knocked down in ACHN cells using small hairpin RNA (shRNA), then the HMGA2-deficient ACHN cells were xenografted into the BALB/c nude mice. Tumor growth was monitored and the expression of EMT-related genes was analyzed.

**Results:**

HMGA2 expression was confirmed to be knocked down in the cultured and xenografted ACHN cells. The xenograft tumor of HMGA2-deficient cells demonstrated a retarded growth pattern compared with the control. The expression of E-cadherin was increased, whereas N-cadherin and Snail were decreased in the HMGA2-deficient xenograft tumors.

**Conclusions:**

In conclusion, to the best of our knowledge, for the first time, we have successfully developed an in vivo experiment using HMGA2-silencing ACHN cells to be grown as xenografts in nude mice. Our findings show that HMGA2 deficiency was sufficient to suppress the xenograft tumor growth in vivo, which support our hypothesis that HMGA2-induced renal carcinogenesis occurs at least in part through the regulation of tumor associated EMT genes.

**Supplementary Information:**

The online version contains supplementary material available at 10.1186/s12885-022-09537-w.

## Background

High-mobility group AT-hook 2 (HMGA2), a member of the high mobility group protein family, is an epigenetic non-histone and architectural transcription regulator and one of the core components of the enhancesome [1]. Through its binding to the AT-rich DNA sequences of promoter regions of target genes and modification of chromatin condensation, HMGA2 alters the transcription of target genes. HMGA2 plays an important role in the embryonic development [1]. Ectopic expression of HMGA2 has been found in various cancers including clear cell renal cell carcinoma (CCRCC), lung adenocarcinoma, colon adenocarcinoma, stomach carcinoma and others [2]. HMGA2 overexpression has also been strongly associated with advanced TNM stage, tumor local invasion and distal metastasis, tumor differentiation and unfavorable prognosis [2]. Mechanistically, recent studies have suggested that HMGA2 may participate in carcinogenesis by regulating the expression of critical genes related to epithelial- mesenchymal transition(EMT), cell proliferation, DNA damage repair and cancer cell stemness [3]. Early studies from our lab showed that HMGA2 is significantly overexpressed in CCRCC specimens of patients [4] and RNAi silencing of HMGA2 gene in cultured renal cell carcinoma (RCC) ACHN cells resulted in decreased cell ability of proliferation and invasion [5]. Another recent study reported that HMGA2 silencing in ACHN cells affected the expression of several EMT-related genes including E-cadherin, N-cadherin, Twist1 and Twist 2, as well as the expression of growth factor TGF-beta and smad2 [6]. However, the studies of HMGA2’s role in renal cancer remain incomplete since most studies were done using in vitro cultured cells and whether the same regulatory pattern also occurring in vivo remains unknown.

Aiming to fill this gap, in the present study, we performed an in vivo experiment with the HMGA2-knockdown ACHN cells by stable RNAi-silencing, which were grown as tumor xenografts in nude mice. Our results show that knockdown of HMGA2 significantly inhibited the tumor growth of ACHN cells and HMGA2 regulates the expression of EMT-related genes in vivo.

## Methods

### Materials

ACHN cells, a renal cell carcinoma cell line, were obtained from the Cell Bank of the Institute of Basic Medical Sciences, Chinese Academy of Medical Sciences, Peking Union Medical College (Beijing, China). The Trizol™ reagent was obtained from Thermo Fisher Scientific (Waltham, MA, USA). Rabbit anti-HMGA2 antibody (Ab207301), Rabbit anti-E- cadherin antibody (Ab40772), Rabbit anti-N-cadherin antibody (Ab76011), Rabbit anti-Snail antibody (Ab216347) were purchased from Abcam (Boston, MA, USA). Goat anti-rabbit IgG-HRP was purchased from KeyGenBioTECH (Nanjing, China). The Reverse Transcription kit and One Step TB Green™ PrimeScript™ RT-PCR Kit II (SYBR Green) were purchased from TaKaRa-Bio (Dalian, China). The primers were ordered from Beijing Aoke Biotechnology Co., Ltd. (Beijing, China). The bicinchoninic acid (BCA) protein assay kit was purchased from Shenyang Wanlei Biological Technology Co. Ltd. (Shenyang, China). Three HMGA2 shRNAs and negative control scrambled shRNA were custom-designed and synthesized by KeyGen BioTECH (Nanjing, China).

### Cell culture

The ACHN cells were grown in RPMI-1640 culture medium (Gibco, Grand Island, NY, USA), supplemented with 10% fetal bovine serum (Thermo Fisher Scientific, Waltham, MA, USA) and 100 μg/ml streptomycin plus 100 U/ml penicillin (Invitrogen, Carlsbad, CA, USA), and cultured at 37 °C under 5% CO2 atmosphere.

### Lentiviral small hairpin RNA vector construction and transfection

Three independent oligoneucleotides for shRNAs specifically targeting HMGA2 gene together with the scrambled control shRNA were designed, synthesized and inserted into the shRNA expression vector U6- MCS-CMV-zsGreen-PGK-Puromycin. The three HMGA2 shRNA target sequences were HMGA2- shRNA-1: 5′-AGTCCCTCTAAAGCAGCTCAA-3′, HMGA2-shRNA-2: 5′-CCGAATTGGGTTTAGTCAATC-3′ and HMGA2-shRNA-3: 5′-AGGAGGAAACTGAAGAGACAT-3′. For synthesis of the shRNAs, the following primers were used shRNA1 Forward: 5′- GATCAGTCCCTCTAAAGCAGCTCAACTCGAGTTGAGCTGCTTTAGAGGGACTTTTTTT-3′, shRNA1 Reverse: 5′-AATTAAAAAAAGTCCCTCTAAAGCAGCTCAACTCGAGTTGAGCTGCTTTAGAGGGACT-3′, shRNA2 Forward: 5′- GATCCCGAATTGGGTTTAGTCAATCCTCGAGGATTGACTAAACCCAATTCGGTTTTTT-3′, shRNA2 Reverse: 5′-AATTAAAAAACCGAATTGGGTTTAGTCAATCCTCGAGGATTGACTAAACCCAATTCGG-3′, shRNA3 Forward: 5′- GATCAGGAGGAAACTGAAGAGACATCTCGAGATGTCTCTTCAGTTTCCTCCTTTTTTT-3′, shRNA3 Reverse: 5′-AATTAAAAAAAGGAGGAAACTGAAGAGACATCTCGAGATGTCTCTTCAGTTTCCTCCT-3′.

After annealing, the oligonucleotides were incorporated downstream from the U6 promoter in the lentiviral vector pLenti-CMV-zsGreen–PGK-Puromycin (KeyGen BioTECH, Nanjing, China). The scrambled control shRNA was inserted into the lentivirus as non-specific control. For stable transfection, ACHN cells were treated with viral supernatant and Polybrene and incubated for 8 h. The cells were selected with puromycin (2 μg/ml) for 7 days. The knockdown of HMGA2 in transient transfected cells (with HMGA2 siRNA1, siRNA2, siRNA2), in stable transfected cells (with HMGA2 shRNA2) and in xenograft tumor (with shRNA2) were confirmed with RT-PCR and western blotting.

### Animal experiments

To obtain the in vivo tumorigenesis model, 4 X 10^6^ ACHN cells, grown in culture medium and harvested in mixed population (Group 1: ACHN cells without transfection; Group 2: ACHN cells transfected with scrambled shRNA; Group 3: ACHN cells transfected with HMGA2 shRNA; 4 animals per group to satisfy the minimum sample size required for statistical analysis) in a volume of 0.2 ml were injected subcutaneously into the right axillary region of 4 weeks old female BALB/c nude mice (SLAC Laboratory Animal Ltd., Shanghai, China). The xenograft tumors could be observed 18 days after injection. The tumor sizes were monitored every other day for 30 days and the tumor volume was calculated in mm^3^ as (width)^2^ x length/2. The mice were maintained with standard care and food/water supply and euthanized using CO_2_ inhalation. The total body weight was recorded at the time of necropsy.

### Reverse transcription and quantitative-polymerase chain reaction (RT-qPCR)

Total RNA extraction was performed using the Trizol™ method according to manufacturer’s instruction. Subsequently, 2μg of total RNA was reverse transcribed into cDNA. For PCR amplification, the following primers were used: HMGA2 Forward:5′-ACAAGAGTCCCTCTAAAGCAGC-3′; HMGA2 Reverse: 5′-AGGCAACATTGACCTGAGC-3′; GAPDH Forward: 5′-CAAATTCCATGGCACCGTCA-3′; GAPDH Reverse: 5′- AGCATCGCCCCACTTGATTT-3′; E-cadherin Forward: 5′-GTTTACCTTCCAGCAGCCCT-3′; E-cadherin Reverse: 5′-TCCCAGATGAGCATTGGCAG-3′; N-cadherin Forward: 5′- GGCGTTATGTGTGTATCTTCACT-3′; N-cadherin Reverse: 5′-GCAGGCTCACTGCTCTCATA-3′; Snail Forward: 5′-TTCTGTGAGCAGGACATCCG-3′; Snail Reverse: 5′-GCAGCCGTCAATGGCTTTAG-3′. The quantitative PCR was performed using 2× Real Time PCR Master Mix(SYBR Green) on an ABI StepOne plus Real time-PCR system (Thermo Fisher Scientific, Waltham, MA, USA). Quantitative gene expressions of HMGA2, E-cadherin, N-cadherin and Snail were normalized to the expression level of the house-keeping gene GAPDH.

### Western blotting

Cell lysates were harvested from shRNA-transfected ACHN cells or from xenograft tumors using RIPA cell lysis buffer. Total protein concentration was determined using the bicinchoninic acid (BCA) method. Twenty micrograms of protein lysate were separated by 8–12% SDS-PAGE under a constant current of 200 mA for 2 h and protein was transferred onto a PVDF membrane. For blocking of non-specific epitopes, the membrane was incubated in 1X TBS-Tween-20 containing5% (w/v) skimmed milk powder at room temperature for 1 h. Then the membrane was incubated with primary antibodies at 4 °C overnight. The membrane was washed with 1X Tris-based saline-Tween 20 (0.05% v/v) and incubated with horseradish peroxidase-conjugated goat-anti-rabbit IgG at room temperature for 1 h. Then the membrane was washed and developed using ECL reagent (KeyGene BioTECH, Nanjing, China) and the pictures were captured and analyzed using the Chem Image5500 gel imaging system (Alpha Innotec, Santa Clara, CA). The protein expression of HMGA2, E-cadherin, N-cadherin and Snail were semi-quantitatively normalized to the protein expression of GAPDH and expressed as a ratio comparing the band intensities between the two.

### Histological and Immunohistochemical analyses

Formalin-fixed and paraffin-embedded tissues were sectioned at 4 μm. After paraffin sections are routinely deparaffinized to water, antigen retrieval was performed by high temperature ethylene diamine tetraacetic acid (EDTA) solution. All immunohistochemical staining procedures were performed on a Leica ST5010 Autostainer XL (Buffalo Grove, USA). 1:50 mouse anti-HMGA2 monoclonal antibody and horseradish peroxidase-conjugated mouse-anti-rabbit IgG were used as primary and secondary antibodies (Santa Cruz Biotech., USA); DAB was used for color development with hematoxylin counterstaining. The negative control is PBS instead of the primary antibody. HMGA2 protein is positive in brownish-yellow.

### Statistical analysis

Statistics were performed using the SPSS 17.0 statistical software (SPSS Inc., Chicago, IL, USA). In assessment of the significance, the experimental group and the control group were compared using a Student’s t-test or (nonparametric one-way ANOVA) Kruskal- Wallis test. *P* value less than 0.05 was considered to be statistically significant. The power of study was set at 0.8. To set the sample size for the animal experiment, a thumb rule was following as “sample size = 2 SD^2^ (1.96 + 0.842)^2^/d^2^”. The criteria were set as type I error *p* = 0.05 and type II error with power = 0.8. Since the experiment used xenograft tumors with HMGA2- knockdown ACHN cells, the standard deviation was expected to be relatively small between 10 and 15%, therefore a sample size of 4 animals allows detection of observational difference (changes of EMT marker expression) (d)≧20% (if SD = 10%), or ≧30% (if SD = 15%).

## Results

### Validation of HMGA2 silencing in ACHN cells

**Fig. 1:**
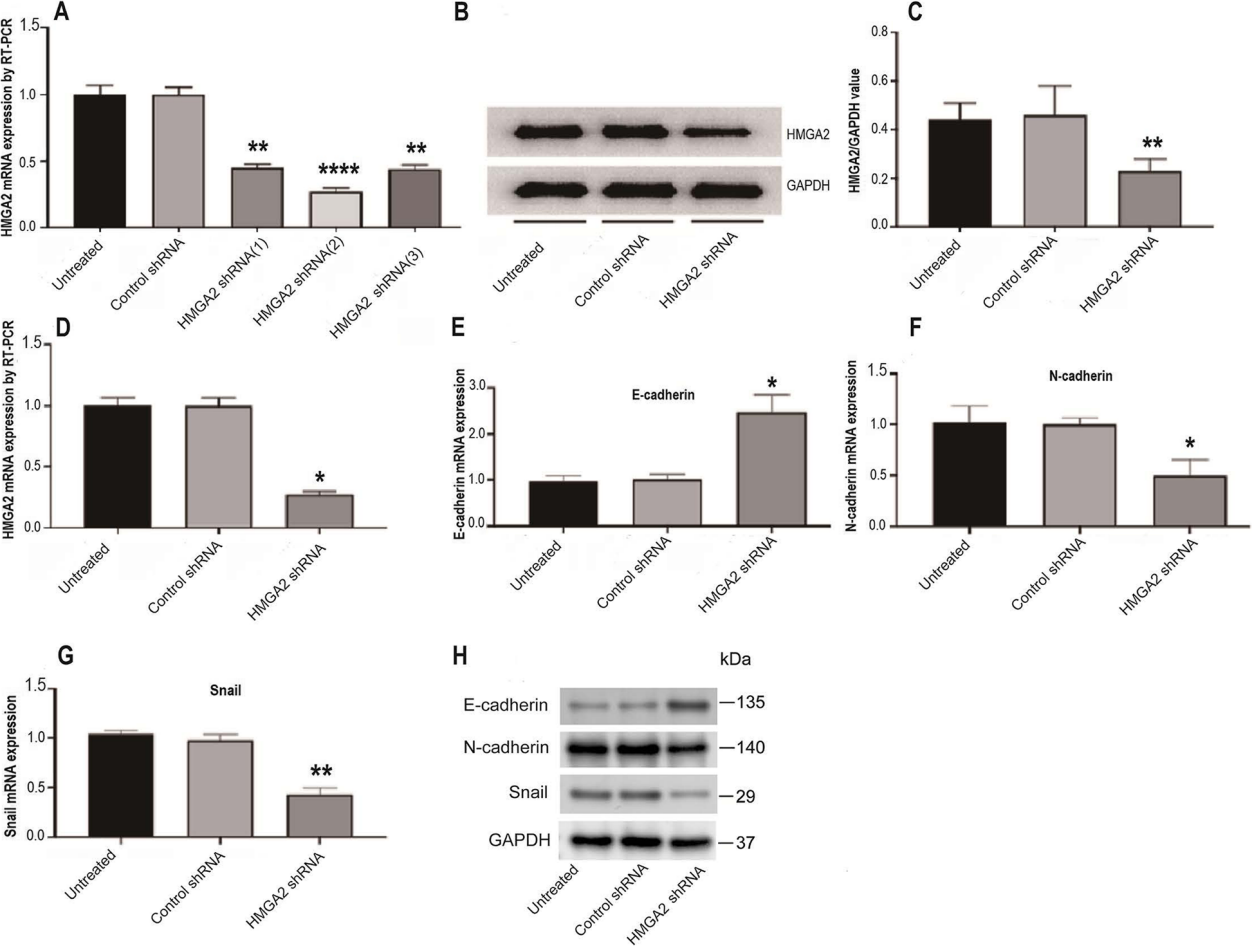
RT-PCR and western blotting results validated the efficacy of shRNAs to silence the expression of HMGA2 gene in ACHN cells. **A** RT-PCR analysis of the HMGA2 mRNA expression in ACHN cells receiving either a scrambled control shRNA or a shRNA (#1-#3) specific to HMGA2 gene (statistical significance with *p* = 0.002, *p* < 0.001, *p* = 0.002 for the three shRNA 1–3). **B** Western blotting analysis of the HMGA2 and GAPDH protein expression in ACHN cells receiving HMGA2 shRNA2. **C** Quantification was performed by normalizing the protein expression level of HMGA2 against the level of the house-keeping gene GAPDH (*p* = 0.002**). **D** the mRNA expression level of HMGA2 in stable-transfected and puromycin-selected ACHN cells (*p* = 0.013*). This is done before xenograft modeling. **E-G** The mRNA expression level of EMT markers E- cadherin, N-cadherin and Snail in cultured ACHN cells (*p* = 0.0132 *, *p* = 0.0102 *, *p* = 0.0024 **, for the three groups). **H** Western blot results for protein expression of EMT-related genes in HGMA2-silenced ACHN cells before xenografting. Data were expressed as mean +/− standard deviation. For each group, data were obtained from three independent repeat experiments, with triplicate samples in each repeat. Statistical significance was calculated using Kruskal-Wallis test

Upon the shRNA transfection in ACHN cells, the knockdown of HMGA2 expression was confirmed by RT-PCR and Western blotting. As shown in Fig. 1A, 24 h after transfection, HMGA2- shRNA1, shRNA2, shRNA3 significantly reduced HMGA2 mRNA expression to 45, 27 and 44% relative to the level as in the untreated cells, but the scrambled control shRNA did not show significant change. Therefore, it was confirmed that all the shRNAs reduced HMGA2 mRNA expression and shRNA2 has the most significant inhibitory effect. To further validate the knockdown on the HMGA2 protein level, western blotting was used to measure the HMGA2 protein expression in cells treated with shRNA2. As shown in Fig. 1B and C, the protein expression of HMGA2 in the treated cells decreased to 23% of the level as in the untreated cells. No significant change was found in the scrambled control shRNA-treated cells. Together, these results confirmed that the shRNA successfully silenced the HMGA2 gene expression in ACHN cells, and the shRNA2 with the greatest effect was determined to be used in the further experiments.

### HMGA2 silencing inhibits the xenograft tumor growth

**Fig. 2:**
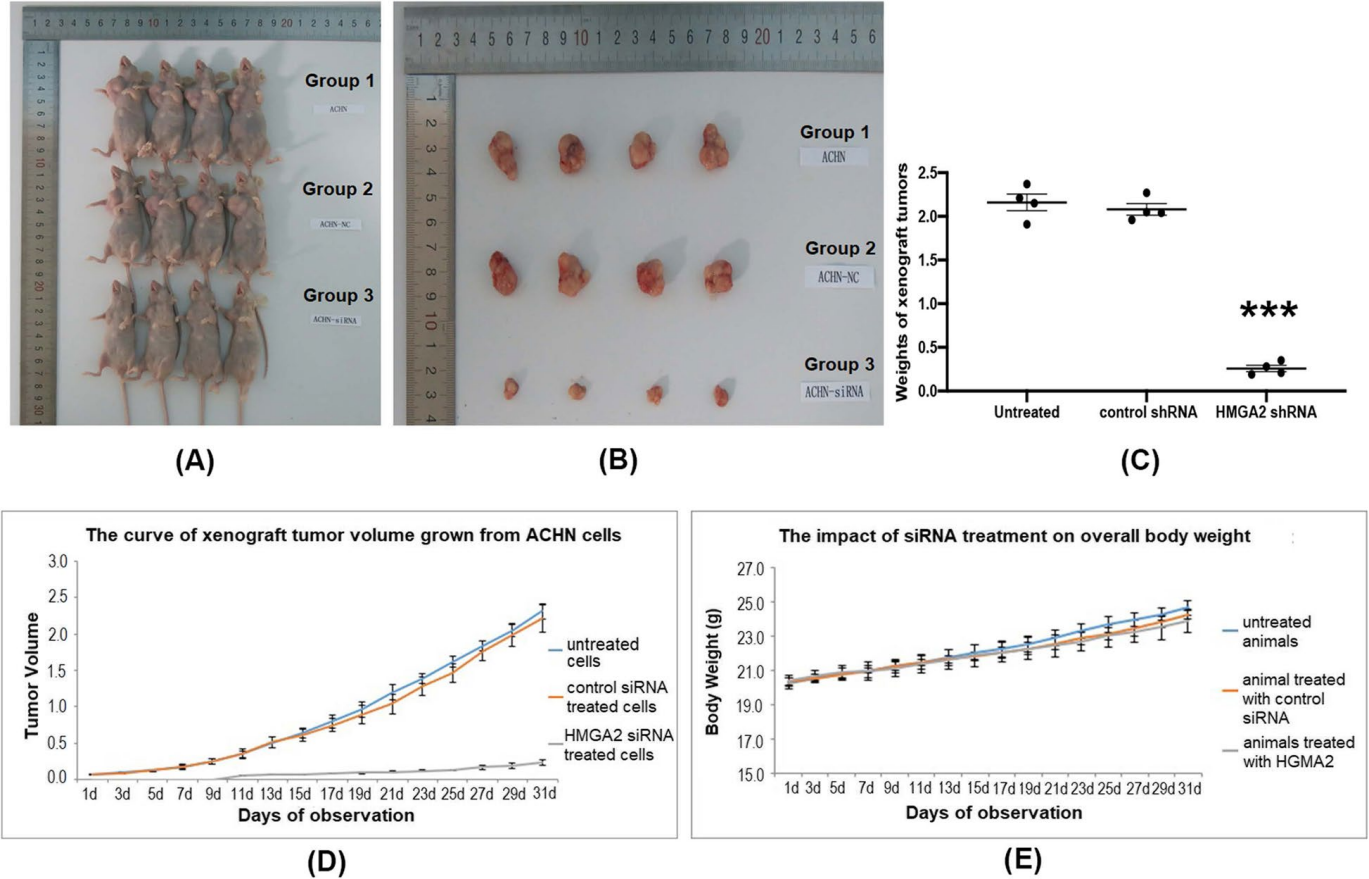
Xenograft tumors of untreated ACHN cells (group 1), ACHN cells treated with scrambled control shRNA (group 2) and cells treated with HMGA2-specific shRNA (group 3) in 4 weeks old female BALB/c nude mice. Tumors began to be observable 18 days after injection of 4 X 10^6^ ACHN cells into the right axillary region of mice and the observation was maintained for 30 days until necropsy. **A** Pictures of nude mice receiving the above described treatments. **B** Pictures of xenograft tumors harvested at the time of necropsy. **C** Quantification of the average tumor sizes of three treatment groups. **D** Growth curves of xenograft tumors with size being recorded each other day for 30 days. **E** Curve of the body weight changes of the mice of the three treatment groups

The effect of HMGA2 silencing on tumorigenesis was studied by xenografting HMGA2 shRNA-transfected ACHN cells (mass population) into the subcutaneous regions of 4 weeks old female BALB/c nude mice. Before injection, the knockdown of HMGA2 was further confirmed as shown in Fig. 1D. It was also confirmed that knockdown of HMGA2 led to decreased expression of N-cadherin and Snail, and increased expression of E-cadherin gene and protein levels in the cultured ACHN cells (Fig. 1E-H). Three experimental groups were included: one group using untreated ACHN cells (Group 1), another group using scrambled control shRNA-transfected ACHN cells (Group 2), and the third group using HMGA2 shRNA2-transfected ACHN cells (Group 3). Each group contains 4 animals. The xenograft tumors were observable 18 days after injection of the designated ACHN cells (mass population) into the right axillary subcutaneous region. Then the tumor growth was continuously monitored for 4 weeks until necropsy. As shown in Fig. 2A-C, the xenograft tumors from the HMGA2 shRNA2-treated ACHN cells were significantly smaller than those grown from the untreated or scrambled shRNA-treated ACHN cells (*p* < 0.001). The average size of the tumors was 2.16 cm^3^ in group 1, 2.08 cm^3^ in group 2 and 0.26 cm^3^ in group 3, respectively. In addition, the inhibition rate was 88% by HMGA2 silencing (Fig. 2C), and the pattern of growth inhibition was consistent throughout the entire observation period of 30 days (Fig. 2D). Notably, there was no significant change in overall body weight among the three groups (Fig. 2E), indicating that the difference in tumor size was not causatively correlated with the tumor burden or the cachexia condition. These results suggested that HMGA2 plays an important role in tumor cell proliferation in vivo since HMGA2 silencing retarded the growth of xenograft tumors in nude mice.

### HMGA2 silencing altered the expressions of EMT-related genes in xenograft tumor

**Fig. 3:**
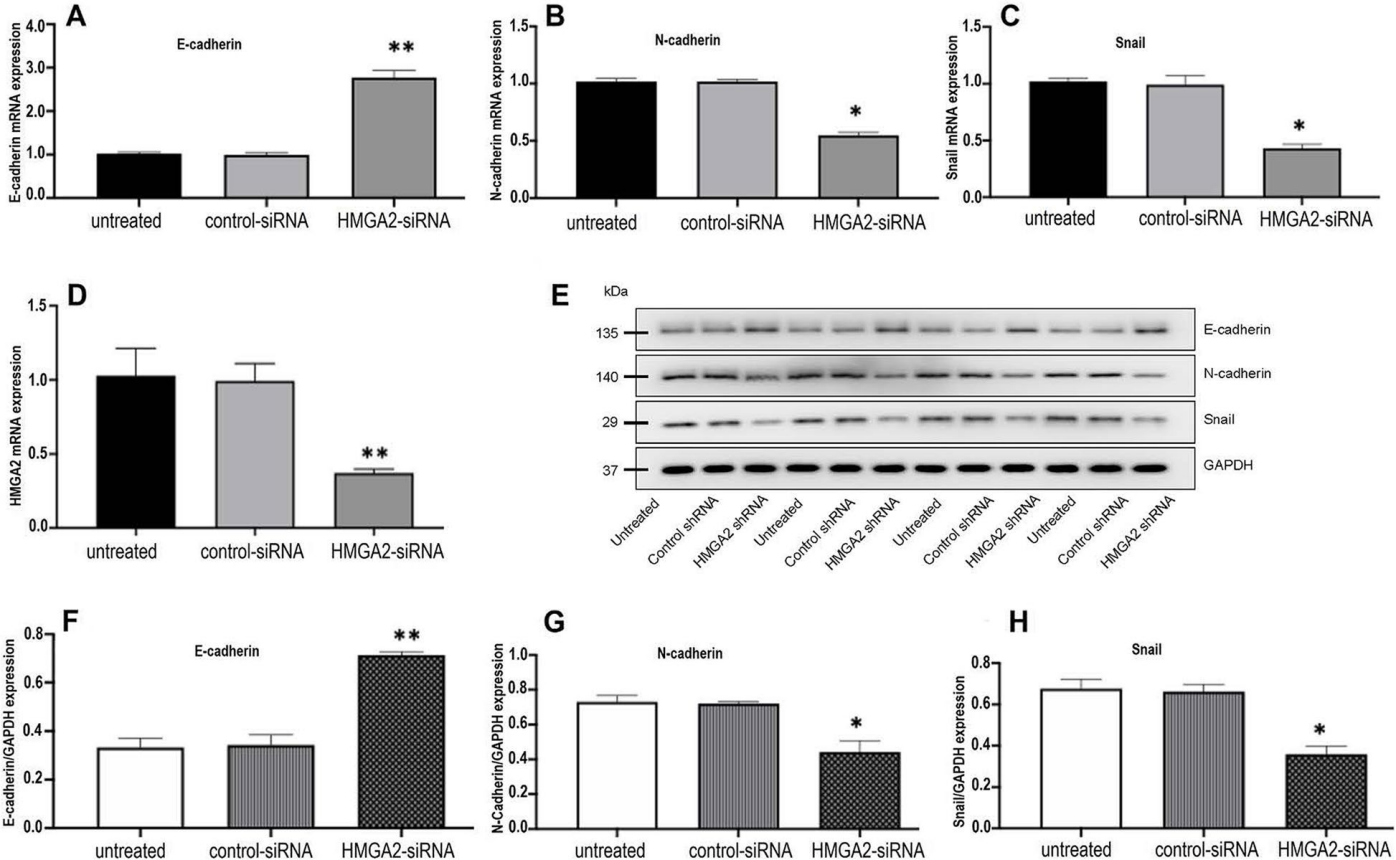
RT-PCR and western blotting analysis of E-cadherin, N-cadherin and Snail expression in xenograft tumors of untreated ACHN cells, ACHN cells treated with scrambled control shRNA and cells treated with HMGA2-specific shRNA. **A-D** mRNA expression of E-cadherin (*p* = 0.002**), N-cadherin (*p* = 0.013*), Snail (*p* = 0.0117*) and HMGA2 (*p* = 0.002**). **E** Western blot gel pictures of E-cadherin, N-cadherin, Snail and GAPDH. **F-H** Quantification of E- cadherin (*p* = 0.007**), N-cadherin (*p* = 0.011*) and Snail (*p* = 0.013*) protein expression under three treatment conditions. Normalization was performed by comparing the protein expression level of the above describe genes against the level of the house-keeping gene GAPDH (statistically significance with *p* < 0.05). Data was present as mean value +/− standard deviation. Statistical significance was calculated using Kruskal-Wallis test

Results from Fig. 2 clearly showed that HMGA2 knockdown significantly inhibited the xenograft tumor growth in vivo, the next effort was to address the molecular mechanism by which the HMGA2 silencing exerting its inhibitory effects on xenografting tumor proliferation. To this end, experiments were carried out to evaluate the mRNA and protein expression of the EMT-related genes including E-N-cadherin, N-cadherin and Snail, which are critical regulators in tumor proliferation. As shown in Fig. 3A-C, the mRNA expression of E- cadherin was significantly increased by ~ 2.6 folds, whereas N-cadherin and Snail expressions were significantly decreased by ~ 2 folds in the HMGA2-silencing xenograft tumors compared to the control group. In addition, results from the western blot confirmed that the protein expression of E-cadherin was upregulated, but the expressions of N-cadherin and Snail were down-regulated in the HMGA2-knockdown xenograft tumors (Fig. 3D-H and Supplementary file S1). Taken together, these results suggest that HMGA2 may inhibit the xenograft tumor proliferation and growth through the regulation of EMT-related gene expressions in the xenograft RCC tumor model.

## Discussion

HMGA2 is a non-canonical epigenetic transcription regulator physiologically expressed in the developing embryonic tissues but ectopically expressed in various human malignancies including renal cell carcinoma [1, 4, 7]. Interestingly, epigenetic regulators are known to play a crucial role in the development of renal cell carcinoma. While inactivation of Von Hippel-Lindau (VHL) occurs in majority of RCC, mutations of PBRM1, SETD2, BAP1 are also common contributors and they are all epigenetic factors [8]. It has been found that PBRM1 modulates HIF1a-VEGF signal and STAT3-Interferon signal [9, 10] and BAP1 promotes genomic instability and therefore accelerates tumor metastasis [11, 12]. Hence, it would be interesting to examine whether, and if so how, the epigenetic factor HMGA2 involves in the pathogenesis of RCC. In our previous studies, we found that HMGA2 was overexpressed in the human RCC specimens, whereas very limited or no expression was presented in benign or adjacent normal tissues [4]. In addition, the level of HMGA2 expression is correlated with the advanced TNM stage and lymph node metastatic status, but not associated with age, gender, tumor size or histology subtypes [4]. Studies from other teams further confirmed that HMGA2 expression was correlated with the overall survival in RCC patients [6]. Therefore, it seems that the aberrant expression of HMGA2 gene links to the malignant and metastatic behavior of RCC.

The molecular mechanism of HMGA2-involved gene regulation in RCC remains to be elucidated though several genes involved in cell proliferation, differentiation, apoptosis, DNA damage and repair, and epithelial-mesenchymal transition had been reported [13, 14]. Previous in vitro studies from our and other groups using ACHN cell lines have shown that HMGA2-knockdown significantly depressed the cell proliferation, metastasis behavior and tumor growth [5, 6]. Another study found that the HMGA2-knockdown ACHN cells increased the expression of E-cadherin and decreased the expressions of N-cadherin, Twist1 and Twist2, whereas gain-of-function of HMGA2 exerted opposite effects in the ACHN cells [6]. These in vitro studies suggested that HMGA2 may regulate the growth and metastasis of RCC cells through EMT.

In the present study, for the first time, we analyzed the tumor growth and a few key EMT markers in the xenograft tumors in vivo, it provides a potential powerful model for future investigations. For example, using the same model, high throughput RNA sequencing techniques would allow us to identify more genes being regulated by HMGA2 (e.g., by HMGA2 shRNA treatment) in cultured ACHN cells as well as in xenografted ACHN tumors. One limitation of our present study is that we analyzed only a small number of regulators as EMT markers in vivo, and additional regulatory genes warrant further investigation. Another limitation of the present study is that only one type of RCC cell line was used and a minimal number of mice with xenograft tumor were investigated. More insights could be gained by analyzing multiple cell lines as well as human specimens in relation to the expression of HMGA2 and EMT-related genes in the future directions.

## Conclusions

In conclusion, to the best of our knowledge, for the first time, we have successfully performed an in vivo experiment using HMGA2-silencing ACHN cells to be grown as xenografts in nude mice. Our findings show that HMGA2-silencing was sufficient to suppress the cell transformation and xenograft tumor growth in vivo, which is consistent with our hypothesis that HMGA2-induced renal carcinogenesis occurs at least in part through regulating tumor associated EMT gene expression. Further characterization of the functional correlation between HMGA2 and HMGA2-mediated EMT target gene regulation will help us understand the tumorigenesis of renal cell carcinoma.

## Supplementary Information

**Additional file 1: Figure S1**. IHC for HMGA2 protein expression in xenograft tumor tissues. A: high expression of HMGA2 protein in group 1 tumor tissue (400x); B: high HMGA2 protein expression in group 2 tumor tissues (400x); C: low expression of HMGA2 protein in group 3 tumor tissues (400x); D: no HMGA2 protein expression in normal kidney tissues of nude mice (400x). Red arrows indicate positive staining of HMGA2 protein. **Figure S2.** Western blot gel for E-cadherin protein expression in HMGA2-silenced ACHN cell line before xenograft. **Figure S3.** Western blot gel for N-cadherin protein expression in HMGA2-silenced ACHN cell line before xenograft. **Figure S4.** Western blot gel for Snail protein expression in HMGA2-silenced ACHN cell line before xenograft. **Figure S5.** Western blot gel for GAPDH expression in HMGA2-silenced ACHN cell line before xenograft. **Figure S6.** Western blot gel for E-cadherin protein expression in HMGA2-silenced xenograft tumor. **Figure S7.** Western blot gel for GAPDH expression in HMGA2-silenced xenograft tumor. **Figure S8**. Western blot gel for N-cadherin protein expression in HMGA2-silenced xenograft tumor. **Figure S9**. Western blot gel for N-snail protein expression in HMGA2-silenced xenograft tumor.

## Data Availability

All data generated or analyzed during this study are included in this published article. Should there be any further requests and questions, the data used and/or analyzed during the current study are available from the corresponding author on reasonable request. All gels/blots used in the figures followed the digital image and integrity policies.

## Abbreviations

HMGA2: High-mobility group AT- RCC Hook 2 renal cell carcinoma
shRNA: Small hairpin RNA
siRNA: Small interfering RNA
EMT: Epithelial-mesenchymal transition
RNAi: RNA interference
RT-PCR: Real Time Polymerase Chain Reaction
BCA: Bicinchoninic acid
TNM: Tumor node metastasis
