# Supplementary material for: Effects of HMGA2 on the epithelial-mesenchymal transition-related genes in ACHN renal cell carcinoma cells-derived xenografts in nude mice
Source: BMC Cancer. 2022 Apr 19;22:421. doi: 10.1186/s12885-022-09537-w (PMC9016978; doi:10.1186/s12885-022-09537-w)
Supplement: Supplementary file 1 — Additional file 1: Figure S1. IHC for HMGA2 protein expression in xenograft tumor tissues. A: high expression of HMGA2 protein in group 1 tumor tissue (400x); B: high HMGA2 protein expression in group 2 tumor tissues (400x); C: low expression of HMGA2 protein in group 3 tumor tissues (400x); D: no HMGA2 protein expression in normal kidney tissues of nude mice (400x). Red arrows indicate positive staining of HMGA2 protein. Figure S2. Western blot gel for E-cadherin protein expression in HMGA2-silenced ACHN cell line before xenograft. Figure S3. Western blot gel for N-cadherin protein expression in HMGA2-silenced ACHN cell line before xenograft. Figure S4. Western blot gel for Snail protein expression in HMGA2-silenced ACHN cell line before xenograft. Figure S5. Western blot gel for GAPDH expression in HMGA2-silenced ACHN cell line before xenograft. Figure S6. Western blot gel for E-cadherin protein expression in HMGA2-silenced xenograft tumor. Figure S7. Western blot gel for GAPDH expression in HMGA2-silenced xenograft tumor. Figure S8. Western blot gel for N-cadherin protein expression in HMGA2-silenced xenograft tumor. Figure S9. Western blot gel for N-snail protein expression in HMGA2-silenced xenograft tumor. [file 12885_2022_9537_MOESM1_ESM.pdf]

**Supplementary Figures**

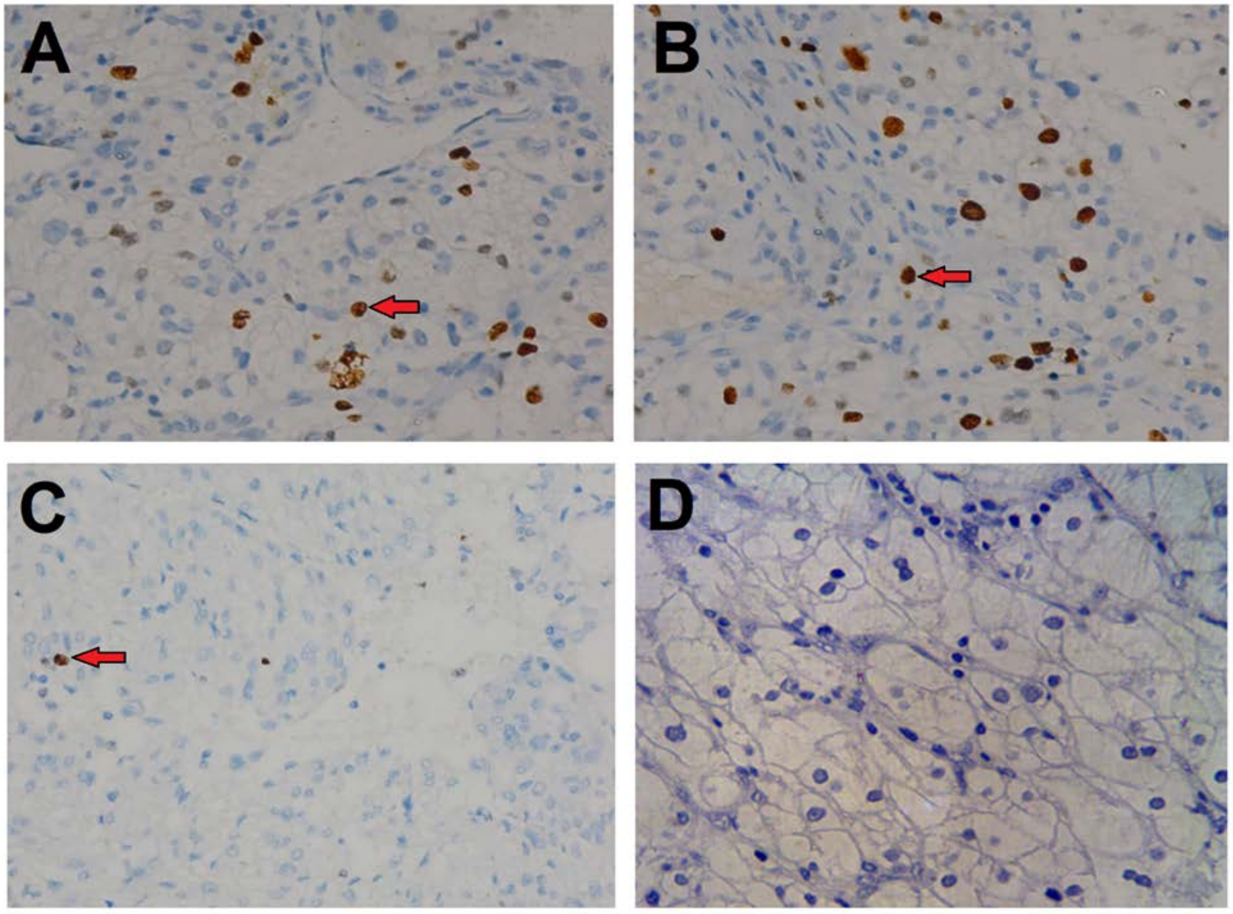


**Figure S1**. IHC for HMGA2 protein expression in xenograft tumor tissues. A: high expression of HMGA2 protein in group 1 tumor tissue (400x); B: high HMGA2 protein expression in group 2 tumor tissues (400x); C: low expression of HMGA2 protein in group 3 tumor tissues (400x); D: no HMGA2 protein expression in normal kidney tissues of nude mice (400x). Red arrows indicate positive staining of HMGA2 protein.

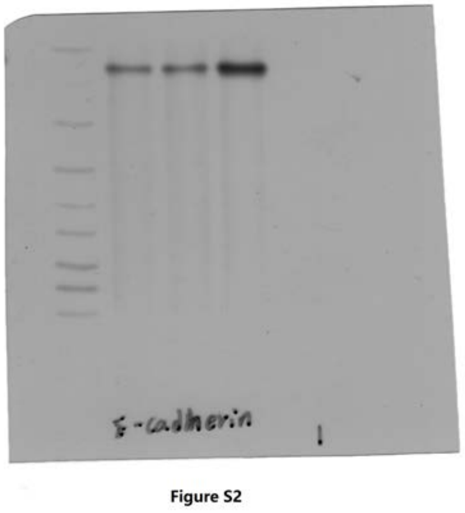


**Figure S2.** Western blot gel for E-cadherin protein expression in HMGA2-silenced ACHN cell line before xenograft.

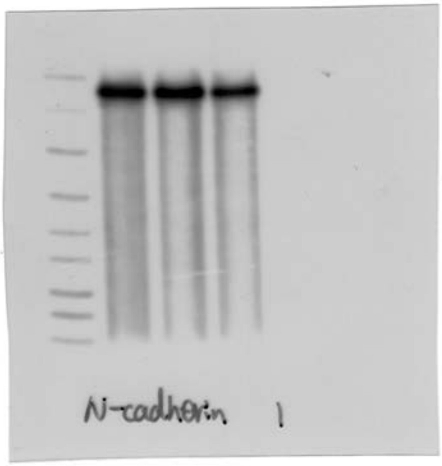


Figure S3

**Figure S3.** Western blot gel for N-cadherin protein expression in HMGA2-silenced ACHN cell line before xenograft.

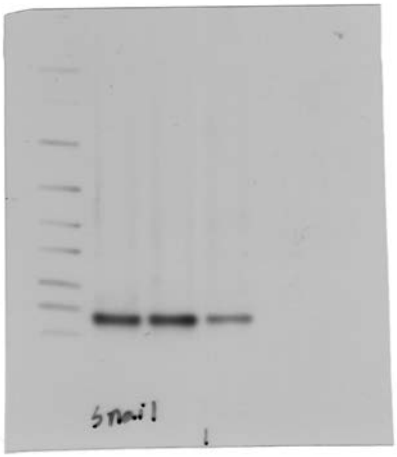


Figure S4

**Figure S4.** Western blot gel for Snail protein expression in HMGA2-silenced ACHN cell line before xenograft.

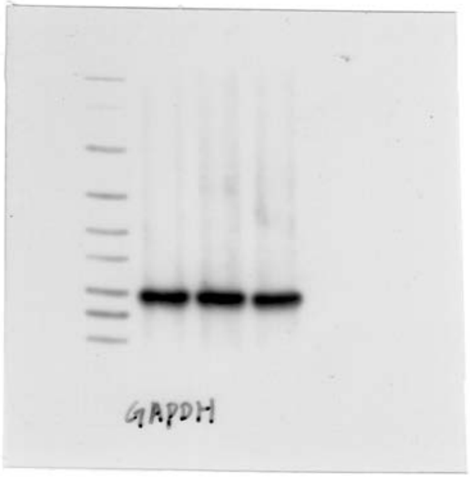


Figure S5

**Figure S5.** Western blot gel for GAPDH expression in HMGA2-silenced ACHN cell line before xenograft.

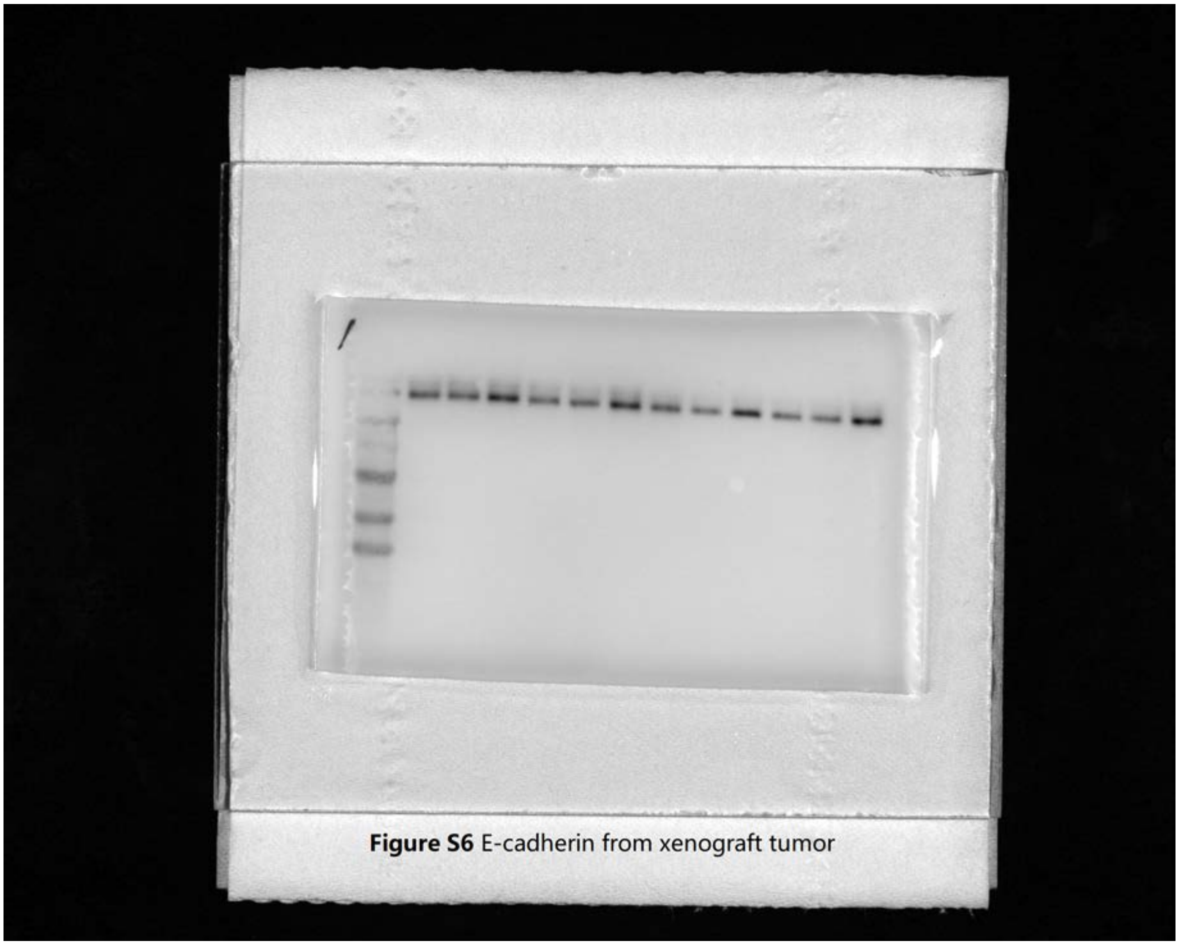


**Figure S6.** Western blot gel for E-cadherin protein expression in HMGA2-silenced xenograft tumor.

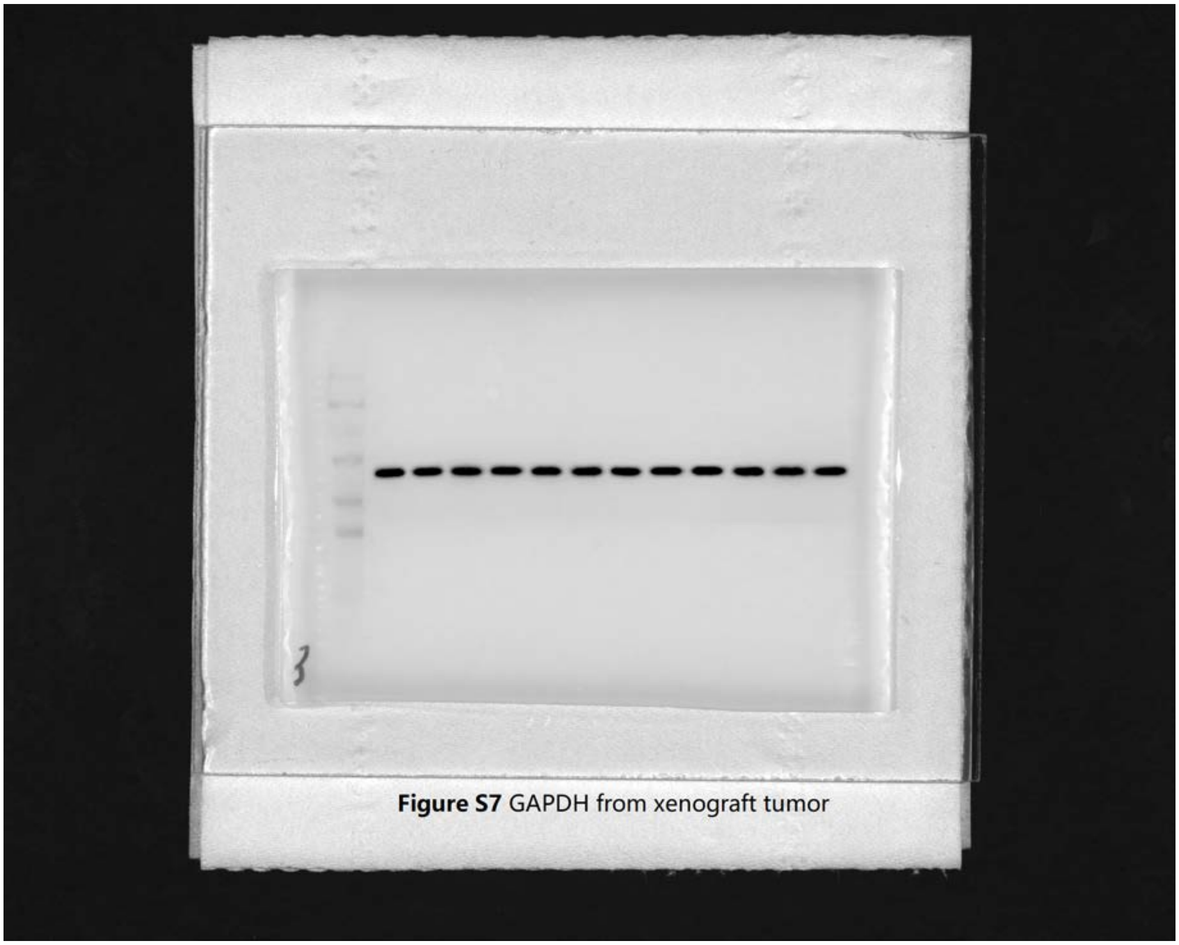


**Figure S7.** Western blot gel for GAPDH expression in HMGA2-silenced xenograft tumor.

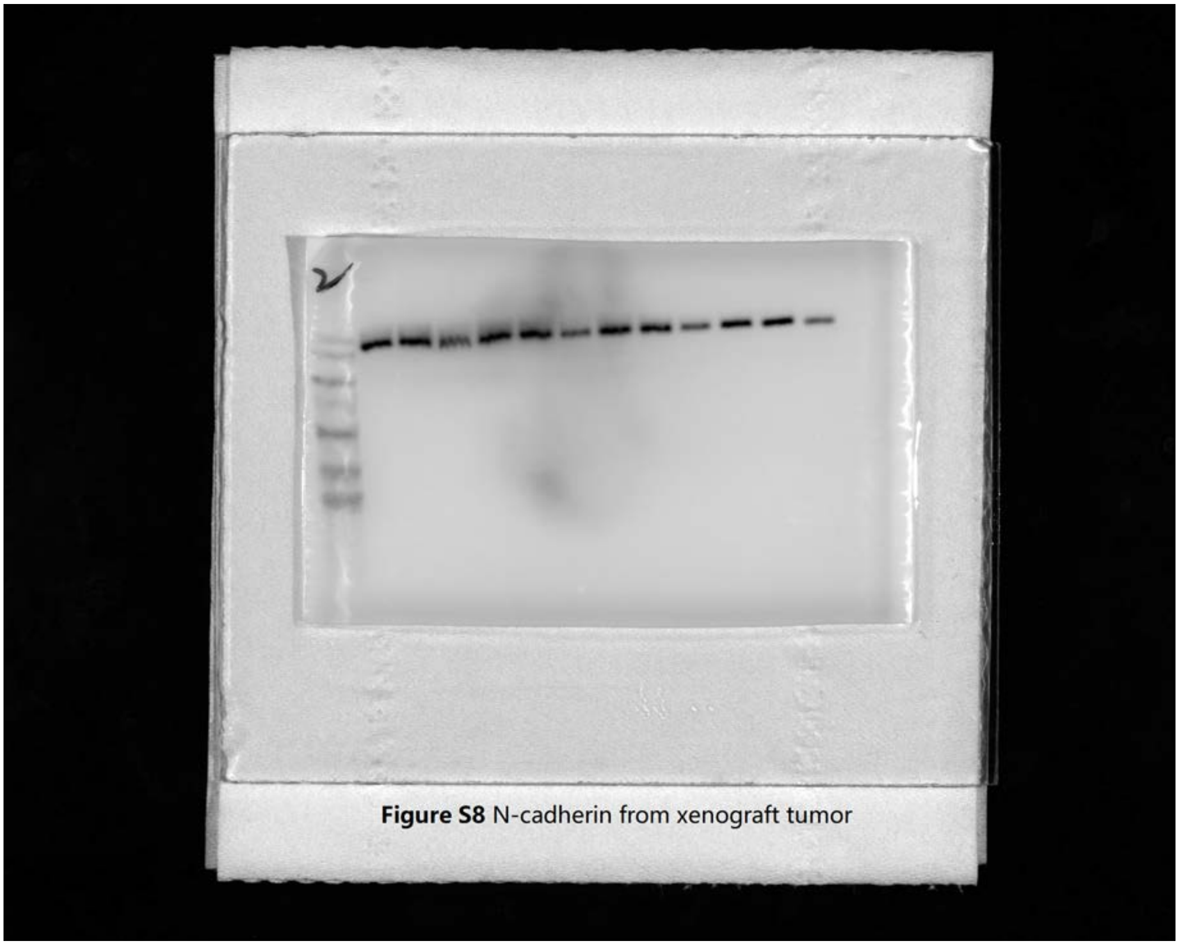


**Figure S8**. Western blot gel for N-cadherin protein expression in HMGA2-silenced xenograft tumor.

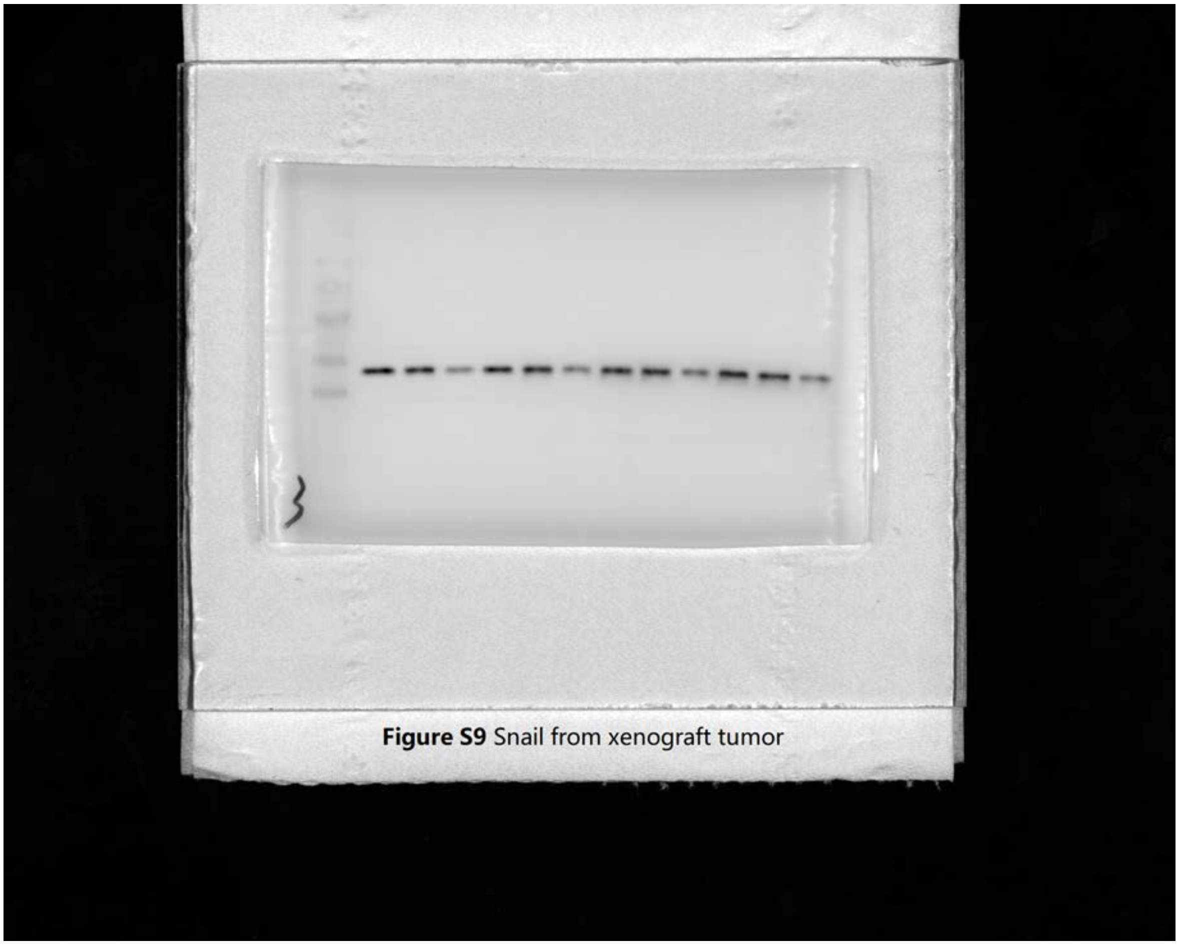


**Figure S9**. Western blot gel for N-snail protein expression in HMGA2-silenced xenograft tumor.
